# Induction of Apoptosis in HeLa Cells by a Novel Peptide from Fruiting Bodies of *Morchella importuna* via the Mitochondrial Apoptotic Pathway

**DOI:** 10.1155/2021/5563367

**Published:** 2021-08-04

**Authors:** Chuan Xiong, Ping Li, Qiang Luo, Chia Wei Phan, Qiang Li, Xin Jin, Wenli Huang

**Affiliations:** ^1^Biotechnology and Nuclear Technology Research Institute, Sichuan Academy of Agricultural Sciences, Chengdu 610061, China; ^2^The Second Affiliated Hospital, Chongqing Medical University, Chongqing 400010, China; ^3^Department of Pharmaceutical Life Sciences, Faculty of Pharmacy, Universiti Malaya, Kuala Lumpur 50603, Malaysia; ^4^Mushroom Research Centre, Universiti Malaya, Kuala Lumpur 50603, Malaysia; ^5^Key Laboratory of Coarse Cereal Processing, Ministry of Agriculture and Rural Affairs, College of Food and Bioengineering, Chengdu University, Chengdu 610106, China

## Abstract

Morels (*Morchella* spp.) are a genus of edible fungi with important economic and medicinal value. In this study, a novel peptide (MIPP) was extracted from the fruiting bodies of *Morchella importuna* using gel filtration chromatography. Structural analysis showed that the molecular mass of MIPP is 831 Da, and it has a simple amino acid sequence: Ser-Leu-Ser-Leu-Ser-Val-Ala-Arg. To explore the antitumor activity of MIPP, the effect of MIPP on HeLa cell apoptosis and the underlying preventative mechanisms were investigated. Results showed that MIPP reduced the viability of HeLa cells in a concentration-dependent manner. TUNEL analysis and flow cytometric examination showed that MIPP decreased cell proliferation via a mitochondrial-dependent pathway, as manifested by downregulation of Bcl-2/Bax, promotion of the movement of cytochrome C from the mitochondria to the cytoplasm, and triggering of caspase-9 and caspase-3. Therefore, MIPP may be a promising tumor-preventive agent, especially in human cervical cancer.

## 1. Introduction

Higher fungi are a very productive source of compounds with biological activity, producing a variety of primary and secondary metabolites. This gives them great potential with regard to drug discovery and development [1]. Studies have confirmed that polysaccharides, polyphenols, flavonoids, triterpenoids, and other bioactive substances can be isolated from fruiting bodies, cultured mycelium, and the mycelial fermentation media of fungi [2, 3]. Specifically, polyphenols [4] and flavonoids [5] derived from fungi are excellent free radical scavenging agents. Fungal polysaccharides are considered to be the most active fungal components, attracting much attention due to their antitumor, antioxidation, and immune regulatory activities [6]. In terms of the nutritional content of mushrooms, we demonstrated that mushrooms are rich in protein, chitin, vitamins, and minerals. However, there is currently little research focused on the efficacy of mushroom proteins or peptides.

Cancer is a leading cause of morbidity and mortality worldwide. Various dietary compounds from natural sources, such as resveratrol, curcumin, isothiocyanates, flavonoids, polyphenols, and peptides, have been shown to suppress the development of cancer cells [7]. Compared with the traditional methods of cancer treatment including surgery, chemotherapy, and radiotherapy, natural therapies tend to have fewer side effects [8]. Resveratrol induced apoptosis in U266 cells via mechanisms completely reliant on Caspase activation and linked with disruption of the mitochondrial membrane [9]. The active flavonoids extracted from *Scutellaria* sp. (skullcaps plant) exhibited antitumor activity via inducing apoptosis and *G*1/*G*2 cell cycle arrest [10]. Apoptosis is a stimulus signal received by the cell in response to changes in environmental conditions or to mitigate cell damage, which results in orderly cell death [11]. The commencement of apoptosis is the beginning and end of a sequence of command switches in the cell after the correlated alert stimulation is detected. Different external factors initiate apoptosis in different ways, resulting in the activation of different signal transduction pathways. As some changes which inhibit apoptosis at tumor progression also limit the reactivity to treatment, apoptosis provides a conceptual foundation connecting cancer genealogy with cancer treatment [12]. Herbs and compounds isolated from natural products have been shown to initiate apoptosis in different human tumor cells and exert antitumor effects, indicating that apoptosis may become an effective target for cancer treatment [13].

Morels (*Morchella* spp.) are a well-known group of edible and medicinal fungi that have a pleasing taste, novel appearance, and economic value, attracting the attention of mushroom researchers worldwide. In Traditional Chinese Medicine (TCM), morels have been proven to exhibit beneficial therapeutic effects on respiratory disorders, excessive phlegm, and indigestion [14]. Further, modern research has confirmed that the polysaccharide from *Morchella esculenta* (MEP) showed notable immune regulatory activity, as it significantly expanded comparative thymus and spleen mass and significantly altered nitric oxide (NO) production [15]. The polysaccharide from *Morchella importuna* (MIP) reduced oxidative damage caused by hydrogen peroxide and exhibited neuroprotective activity [16]. However, it is unknown whether the morel peptides are also biologically active.

The research on peptide drugs is an active field in drug research. Compared to polysaccharides, peptides have a smaller molecular weight and are more likely to pass through structural barriers and enter the interior of the cell. In addition, peptides can be easily synthesized. Thus, the identification of active peptide substances from animals, plants, and microorganisms has become a leading strategy in novel drug research and continues to be a direction in peptide research.

Therefore, the aim of this research was to explore the isolation of morel peptides and evaluate the antitumor activity. In this work, a novel peptide (MIPP) was isolated from the fruiting body of *M. importuna*. It was separated by molecular exclusion chromatography and purified by reversed-phase peak execution fluid chromatography (RP-HPLC), and the sequence was identified by fluid chromatograph-tandem mass spectrometry (LC-MS-MS). The inhibitory effect on HeLa cell proliferation was also evaluated, and the mechanism of HeLa cell apoptosis was explored.

## 2. Materials and Methods

### 2.1. Materials and Chemicals

Fruiting bodies of *Morchella importuna* were obtained from fields owned by the Sichuan Academy of Agricultural Sciences (SAAS). Molecular sequences generated from the fruiting bodies (no. Cyl-158) were deposited at GenBank (http://www.ncbi.nlm.nih.gov) under accession numbers MG121861–MG121865. The fruiting bodies were dried in a 37°C oven. The Sephadex 30 increase column was obtained from GE Healthcare (Uppsala, Sweden). The Brownlee analytical C18 column was bought from Perkin Elmer (Shelton, USA).

The human cervical cancer HeLa cell line was acquired from the West China Medical College, Sichuan University. Dulbecco's modified Eagle's medium (DMEM), fetal bovine serum (FBS), and trypsin-EDTA were from Gibco (Grand Island, NY, USA). 3-(4,5-Dimethylthiazol-2-yl)-2,5-diphenyltetrazolium bromide (MTT) was purchased from Sigma (St. Louis, MO). Kits utilized to determine caspase activity (caspase-3 and -9) were obtained from Beyotime (Shanghai, China). Antibodies for Bax, Bcl-2, caspase-3, cleaved caspase-3, caspase-9, cleaved caspase-9, and GADPH were from Cell Signaling Technology (Danvers, MA, USA). Other chemical reagents involved in this research were of analytical grade.

### 2.2. Segregation and Purification of Peptides

Dried fruiting bodies of *M. importuna* were crushed using a drug pulverizer, and the powder that passed through a 200-mesh screen was collected. The dry morel powder (100 g) was soaked in 2 L of phosphate buffer saline (PBS, pH = 6.8) for 4 hours at room temperature and stirred with a magnetic agitator. The powder was eliminated by medical gauze filtration, and the filtered material was collected. The pooled filtrate solution was separated by centrifuge at 5,000 g for ten minutes to completely eliminate the powder. Soluble protein in the supernatant was obtained by stepwise precipitation of ammonium sulfate ((NH_4_)_2_SO_4_) at a concentration of 40–80%. The precipitate was collected through centrifugation and dissolved in distilled water. The aqueous solution of the precipitate was dialyzed using a dialysis bag (Union Carbide Corp., Houston, TX, USA) with a molecular weight cutoff of 3,500 Da. We concentrated (Eyela, Tokyo, Japan) and lyophilized (Christ, Osterode, Germany) the dialysate to obtain the morel crude protein.

The crude protein extract was added to a Sephadex 30 increase column (2.6 cm × 30 cm). The column was eluted with 5 bed volumes of distilled water at a flow rate of 0.8 mL/min. The eluate was collected (2.5 mL/tube) and detected at 215 nm. We collected the peak with the largest protein content as the protein extract; then, the protein extract was dialyzed, lyophilized, and used for the following experiments.

The protein extract (100 mg) was dissolved in aseptic water and prepared in a 10 mg/mL solution. HPLC analysis was conducted using a Perkin Elmer (Shelton, USA) N2600580 system. A Perkin Elmer Brownlee Analytical C18 column (250 × 4.6 mm) was used at a temperature of 25°C. The loading volume was 30 *μ*L, and the subsequent flow rate was synchronized to 1 mL/min. The mobile stage contained (A) distilled water and (B) acetonitrile trifluoroacetic acid solution (0.1%). Gradient elution consisted of A: 0–8 min, 99%–97%; 8–12 min, 97%–96%; 12–16 min, 96%–80%; and 16–20 min, 80%–99%. The detection wavelength was set to 215 nm. The absorption peak was collected, and the MTT method was used to determine the influence of each component on HeLa cell viability. The component (peptide extract) with the strongest inhibitory effect on HeLa cell viability was selected and lyophilized for the following experiments.

Mass spectrometry analysis was performed using a Triple TOF 5600 LC/MS system (SCIEX, USA). The peptide sample was aspirated through the preprogrammed injector and then merged with the C18 capture column (5 *µ*m, 5 × 0.3 mm); the samples were then eluted and analyzed (75 *μ*m × 150 mm, 3 *μ*m fragment dimension, 100 Å pore expanse, Eksigent). The mobile stage was (A) distilled water trifluoroacetic acid solution (0.1%) and (B) acetonitrile trifluoroacetic acid solution (0.1%). The gradient elution was designed in the following way: 0 min of 3% B, 0.1 min of 3–7% B, 39.9 min of 7–23% B, 3 min of 23–50% B, 2 min of 50–80% B, 5 min of 80% B, 0.1 min of 80–5% B, and 5% B for 9.9 min. The flow rate of the liquid phase was synchronized at 300 nL/min. During the information-dependent acquisition (IDA) mass spectrometry (MS) investigation, one MS full scan was included in each scan cycle (the *M*/*Z* range was 350–1500, and the ion build-up time was 250 ms), accompanied by 40 MS/MS scans (the *M*/*Z* range was 100–1500, and the ion build-up period was 50 ms). The conditions for the MS/MS collection period were set such that the forerunner ion warning was larger than 120 cps, and the charge number was +2–+5. The obtained mass spectrum data was retrieved via ProteinPilot (V4.5).

### 2.3. Morphology Observation

The morphology of the peptide was observed by a scanning electron microscope (SEM), transmission electron microscope (TEM), and atomic force microscope (AFM). The sample (2 mg of the peptide powder) was secured on the SEM reinforcement and sputter-covered with gold under a vacuum using a sputter coater (Q150 R Plus, Quorum, UK) and then microscopically evaluated using an SEM (JSM-7500F, JEOL, Japan) [17]. The samples were examined at an accelerating voltage of 15.0 kV. Representative micrographs were taken of the peptide at ×1000 and 4000 magnification.

The peptide was observed by transmission electron microscopy (TEM) with a Hitachi H-7650 electron microscope. The polypeptide was prepared with Milli-Q water to a concentration of 1 mg/mL; then, the peptide solution and 1% phosphotungstic acid solution were immersed in a copper grid covered with a layer of Formvar membrane. The liquid came into contact with the copper mesh for approximately 10 s and then the mesh was blotted dry with filter paper. The copper grid was further air-dried and then directly observed under an electron microscope.

The peptide was mixed with Milli-Q water to a concentration of 1 mg/mL, and a 10 *µ*L peptide solution was uniformly added to a newly split mica platform. Then, the mica platform was gently cleansed with Milli-Q water to expel the untarnished peptide. The sample was then air-dried and imaged immediately with an AFM (Hitachi SPM400, Japan).

### 2.4. Cell Culture and Treatment

HeLa cells were maintained in Dulbecco's modified Eagle's medium (Gibco Co., USA) supplemented with 10% fetal bovine serum (FBS) in an incubator at 37°C with 5% carbon dioxide. The cells were seeded in a 96-well plate at a density of 3 × 10^3^ cells/well and cultured for 24 hours. The cells were then treated with various concentrations (12.5, 25, 50, 100, 200, and 400 *μ*g/mL) of MIPP (100 *μ*L/well) in culture medium and incubated for 12 hours. The negative and positive control wells contained fresh medium (100 *μ*L/well) or resveratrol (50 *μ*M and 100 *μ*L/well), respectively. Cell viability was measured via the MTT technique. We removed the supernatant and added 10 *μ*L of MTT solution (5 mg/mL in phosphate-buffered saline) and 90 *μ*L of FBS-free medium to every well. Four hours later, 150 *μ*L of dimethyl sulfoxide (DMSO) was added to dissolve the formazan. The rate of absorption of each well was measured at 570 nm in a microplate reader (SpectraMax Plus 384, Molecular Devices, San Jose, CA, USA). The inhibitory rate was calculated using the following equation:

(1) $$\begin{array}{c} \text{inhibitory rate }\left(\%\right)=\;\frac{1-A_{\text{sample}}}{A_{\text{control}}}\;\times 100\%, \end{array}$$

where *A*_sample_ was the absorbance of the cells treated with MIPP or resveratrol and *A*_control_ was the absorbance of the cells treated with fresh medium.

### 2.5. Measurement of Apoptosis

The HeLa cells were grown in 6-well dishes at a density of 3 × 10^4^ cells/well for 12 hours and then treated with various concentrations (50, 100, and 200 *μ*g/mL) of MIPP for 12 hours. After treatment, apoptotic cells were identified using a commercial kit (Invitrogen, China). The experiment was carried out according to the manufacturer's instructions. In general, HeLa cells were digested with trypsin and stained using Annexin V/PI for 15 min. Then, HeLa cells were examined using a FACS Calibur Flow Cytometer (BD, San Jose, CA, USA) at an excitation wavelength of 488 nm and a discharge wavelength of 530 nm. At least 10,000 events were recorded.

### 2.6. Terminal Deoxynucleotidyl Transferase-Mediated dUTP Nick End Labeling (TUNEL) Assay

DNA fragmentation of HeLa cells was observed using TUNEL analysis [18]. The HeLa cells were cultivated in a 6-well dish at a density of 3 × 10^5^ cells/well for 12 hours and then treated with various concentrations (50, 100, and 200 *μ*g/mL) of MIPP for 12 hours in a 5% of CO2-humidified incubator. The medium was discarded, and the cells in each well were washed with PBS three times. Then, the cells were fixed with 4% paraformaldehyde within an hour and permeabilized in a permeabilization solution (0.1% Triton X-100 and 0.1% sodium citrate) for two minutes on ice. The cells were resuspended in 200 *μ*L PBS containing 10 *μ*L of TUNEL reaction compound and incubated at 37°C for one hour. The staining was observed using an inverted fluorescence microscope (DM1000, Leica, GER).

### 2.7. Mitochondrial Depolarization

The change in mitochondrial membrane potential was measured via 5,5,6,6-tetrachloro-1,1,3,3-tetraethyl-imidacarbocyanine iodide (JC-1). The cells were seeded in a 96-well microtiter dish at a density of 3 × 10^3^ cells/well and cultivated for 12 hours. Different concentrations (50, 100, and 200 *μ*g/mL) of MIPP were added to the cells for an additional 12 hours, and cells were then suspended in warm PBS. JC-1 was added to the cells to a final concentration of 2.5 *μ*g/mL per well for 15 minutes. Excitation of FL1 results in red fluorescence and FL2 fluorescence green. The fluorescence intensity was recorded using a FACS Calibur flow cytometer (CytoFLEX, Beckman, USA). The proportion of JC-1 aggregate (FL2, red) to monomer (FL1, green) strength was obtained. This change in proportion is related to mitochondrial depolarization and indicates changes in mitochondrial membrane potential [19].

### 2.8. Determination of Cytochrome C Release

Western blotting was utilized to detect the release of cytochrome C from the mitochondria into the cytosol [20]. HeLa cells were cultured in 6-well plates at a density of 3 × 10^5^ cells/well for 12 hours and then treated with various concentrations (50, 100, and 200 *μ*g/mL) of MIPP for 12 hours in a 5% of CO_2_-humidified incubator. Next, the cells were rinsed twice with precooled PBS buffer. Mitochondria and cytosolic protein extracts were obtained using a commercial kit (Cytochrome C Releasing Apoptosis Assay Kit, Bio Vision, CA, USA). In brief, the cells were bathed in 1.0 mL of 1 × Cytosol Extraction Buffer Mix containing DTT and protease inhibitors and then incubated on ice for 10 minutes followed by gentle homogenization. The homogenate was transferred to a 1.5 mL microcentrifuge tube and centrifuged at 700 × g for 10 minutes at 4°C. The supernatant was collected in a fresh 1.5 mL tube and centrifuged at 10,000 × g for 30 min at 4°C. The supernatant was collected as the cytosolic fraction. Then, the cytosolic fraction was loaded on a 12% SDS-PAGE gel and analyzed via standard Western blotting methods. Blots were analyzed using a cytochrome c antibody (Cytochrome C Rabbit mAb #4280, CST, MA, USA).

### 2.9. Real-Time PCR

Cells (3 × 10^6^) were plated onto 60 mm Petri dishes, and the pretreatment was the same as the above procedure (Section 2.8). TRIzol reagent was used to extract RNA from cells. Primers for Bax, Bcl-2, and GAPDH were designed and synthesized by Tsingke (Guangzhou, China). The specific sequences of primers were as follows: Bcl-2 (F: 5′-ATCTTCTCCTTCCAGCCTGA-3′; R: 5′-TGCAGCTGACTGGACATCTC-3′), Bax (F: 5′-CTGCAGAGGATGATTGCTGA-3′; R: 5′-GAGGAAGTCCAGTGTCCAGC-3′), and GAPDH (the control gene, F: 5′-CACTCACGGCAAATTCAACGGCA-3′; R: 5′-GACTCCACGACATACTCAGCAC-3′). The real-time RT-PCR amplifications were done using SYBR Premix Ex Taq according to the manufacturer's instructions. The real-time PCR operating conditions were as follows: 94°C for 8 min, then 30 cycles at 94°C for 20 s, 56°C for 50 s, and 72°C for 90 s. Finally, incubation was done at 72°C for 3 minutes. The results are expressed as the ratio of optimal density to GAPDH expression.

### 2.10. Analysis of Caspase Activity

Caspase-9 and caspase-3 activity was analyzed using commercial colorimetric assay kits and based on the manufacturer's protocols. After co-culture with MIPP, the HeLa cells were collected and suspended for 5 min in lysis buffer on ice. The supernatant was then collected by centrifugation, and absorbance was read at a wavelength of 405 nm using a microplate reader (Molecular Devices, USA).

### 2.11. Western Blotting

Cells (3 × 10^6^) were plated on 60 mm Petri dishes, and the pretreatment was the same as the above procedure (Section 2.8). The HeLa cells were collected and lysed in NP-40 lysis buffer with protease inhibitors. Protein concentration was determined using the Bradford method. Cell lysates were separated on 12% SDS-PAGE gels and then transferred to polyvinylidene difluoride (PVDF; Bio-Rad) membranes. Then, blocking buffer (Tris-buffered saline containing 3% BSA, 0.2% Tween 20, 2 mM EDTA, and 20 mM NaF) was used to block membranes at room temperature for 1 hour. The membranes were then incubated with the primary antibodies at a dilution of 1 : 1000 at 4°C for 12 hours. Lastly, the secondary antibodies (1 : 3000) were incubated for 1 h, and an ECL™ Western blotting analysis system was used to analyze the data.

### 2.12. Data Analysis

Each experiment was conducted in triplicate. Data obtained is expressed as the mean ± standard derivation (SD) and was evaluated using SPSS 19.0 software (SPSS Inc., Chicago, IL, USA). One-way analysis of variance (ANOVA) was applied to determine significant differences in the categories followed by Dunnett's test. *P* < 0.05 is considered statistically significant.

## 3. Results

### 3.1. Isolation and Purification of MIPP

**Figure 1:**
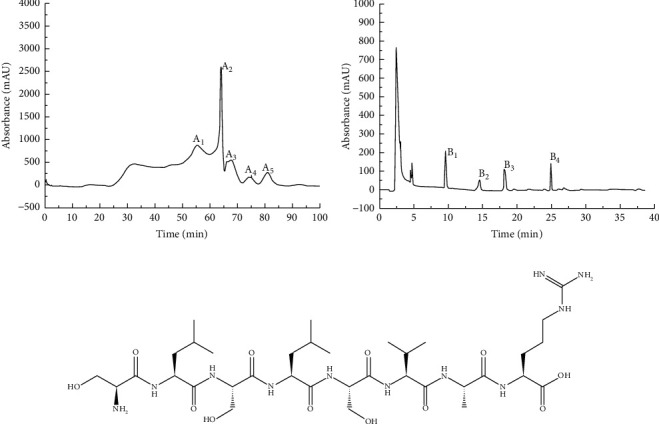
Isolation and purification of MIPP. (a) Gel chromatography. (b) RP-HPLC. (c) Amino acid sequence of MIPP. The crude protein extract of *M. importuna* was obtained by ammonium sulfate precipitation. Sephadex-30 chromatography was used to purify the crude protein extract, and five characteristic absorption peaks were obtained (A1–5). The A3 component was further separated by RP-HPLC, and four fine components were obtained (B1–4). The B4 component was further analyzed by LC-MS-MS to determine the amino acid composition of MIPP.

The crude protein extract of *M. importuna* was obtained by stepwise ammonium sulfate precipitation using a dialysis bag. Extract (16.8 g) was obtained from 200 g of dry powder from morel fruiting bodies with a yield of 8.4%. The crude protein extract was separated and fractionated on a Sephadex 30 increase column (2.6 cm × 30 cm) and washed with distilled water. After 3 times of bed column volume, five elution peaks (A1-5) were obtained (Figure 1(a)). A3 was selected as the morel protein extract and further separated by RP-HPLC (Figure 1(b)). After separation by RP-HPLC, four characteristic absorption peaks were identified (B1-4). Finally, the *M. importuna* (MIPP) peptide was obtained by LC-MS-MS analysis of the B4 component. The relative molecular weight of MIPP was 831 Da, and the compound consisted of 8 amino acids. The amino acid sequence composition was as follows: Ser-Leu-Ser-Leu-Ser-Val-Ala-Arg (Figure 1(c)).

### 3.2. MIPP Morphology Observation

**Figure 2:**
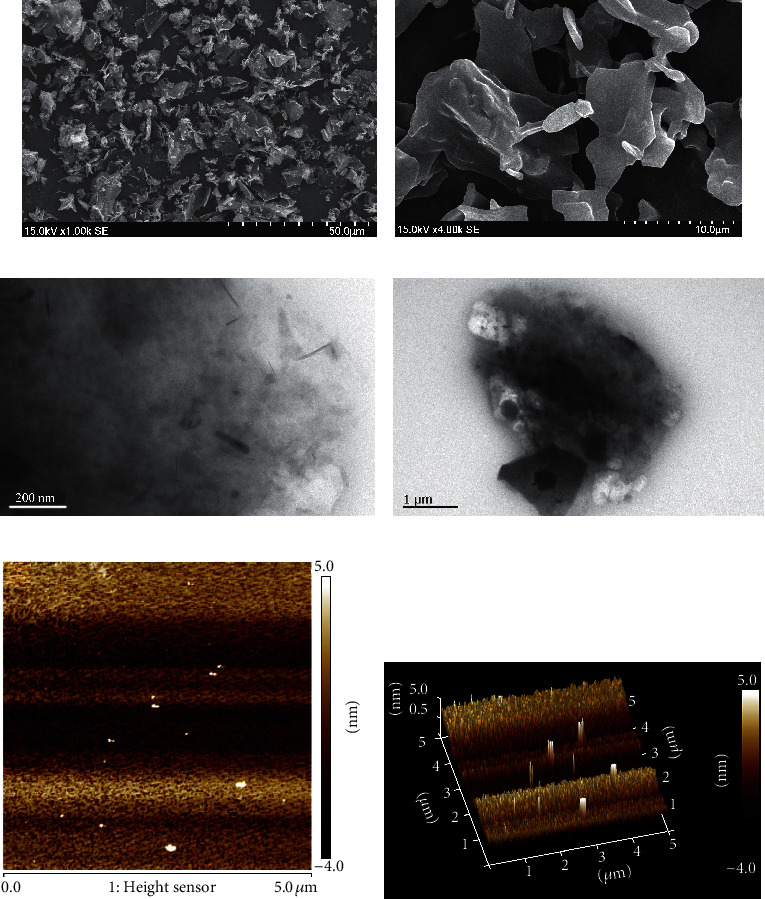
Morphology of MIPP. (a), (b) Scanning electron micrograph images. (c), (d) Transmission electron microscope images. (e), (f) Atomic force microscopy image, E for the 2D image and F for the 3D image.

As revealed by SEM, MIPP had an irregular flaky structure (Figure 2(a)) with a smooth surface (Figure 2(b)). TEM results also showed that MIPP has a tiny sheet structure (Figures 2(c) and 2(d)). As shown in Figure 2(e), in an aqueous solution, MIPP presents a tight, granular arrangement. More than 80% of the particles had a height of 3–5 nm and the tip of the particle was pointed (Figure 2(f)). The average height of the particle tip was 1.38 nm.

### 3.3. The Effect of MIPP on HeLa Cell Proliferation

**Figure 3:**
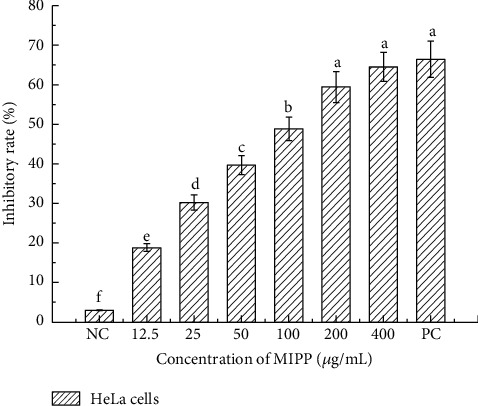
The effect of MIPP on HeLa cell proliferation. Inhibitory effects on HeLa cells were measured by MTT assay following treatment with different concentrations of MIPP for 24 h. Data are expressed as means ± SD (*n* = 3). Values with different superscript letters were significantly different from each other at *P* < 0.05. NC: negative control, cells cultured with 10% FBS high glucose DMEM complete medium; PC: positive control (50 *μ*M resveratrol).

The effect of MIPP on HeLa cell proliferation is shown in Figure 3. When HeLa cells were treated with MIPP at a concentration of 12.5 *μ*g/mL for 12 h, cell proliferation was inhibited 18.83% ± 0.98 compared to controls. With increasing concentrations of MIPP, the inhibition rate of HeLa cell proliferation further increased in a dose-dependent manner. The inhibition rate of 200 *μ*g/mL MIPP was 59.36 ± 3.67% and at 400 *μ*g/mL it was 61.87 ± 3.49%, which was close to the inhibition rate of the positive control resveratrol (50 *μ*M, 63.21 ± 4.58%). Additionally, the inhibition rates of MIPP at 200 *μ*g/mL and 400 *μ*g/mL were not significantly different from each other. Therefore, in subsequent experiments, the upper limit of MIPP concentration was set at 200 *μ*g/mL.

### 3.4. MIPP-Induced Apoptosis in HeLa Cells

**Figure 4:**
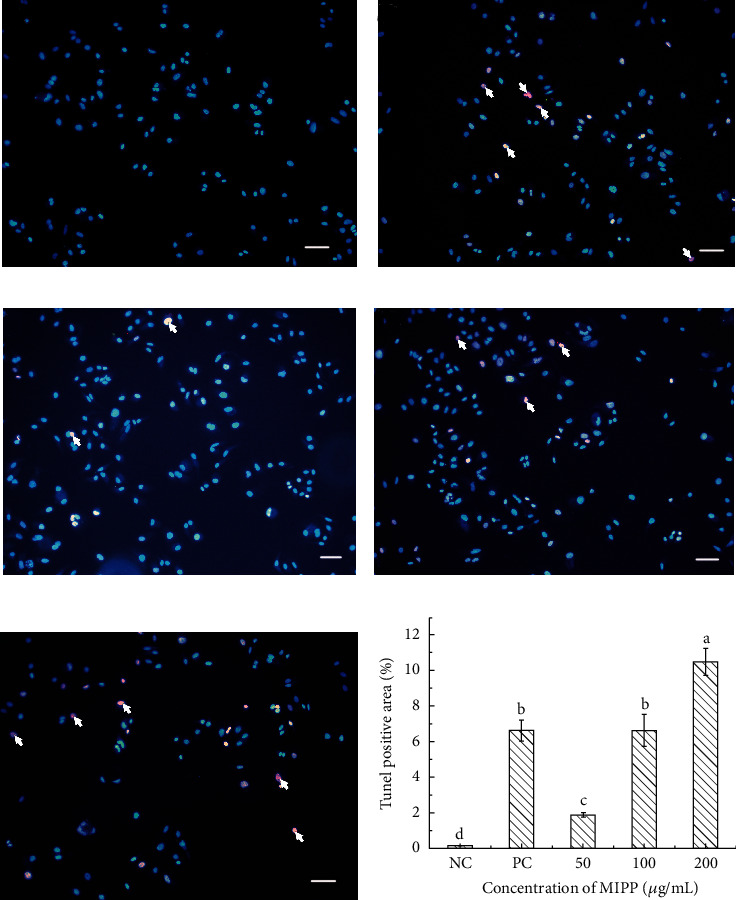
Nuclear morphological changes induced by MIPP in HeLa cells determined via TUNEL assay. (a) Cells cultured under standard conditions (negative control). (b) Cells treated with resveratrol (50 *μ*M) as the positive control. (c), (d), and (e) Cells treated with different concentrations (50, 100, and 200 *μ*g/mL) of MIPP. (f) Semiquantitative analysis of TUNEL positive area of HeLa cells from immunofluorescence staining. The HeLa cells were cultivated under standard conditions for 12 h before treatment with different concentrations (50, 100, and 200 *μ*g/mL) of MIPP and resveratrol (50 *μ*M) for 12 h 4′,6-diamidino-2-phenylindole (DAPI) excites blue fluorescence in the nuclei of cells. TUNEL reaction mixture shows apoptotic cells via red fluorescence. In the superimposed image, the nuclei of apoptotic cells exhibit a light purple fluorescence. The arrow in the figure points to the nuclei of apoptotic cells. Scale bar = 50 *μ*m. The semiquantitative analysis was carried by Image Pro Plus 6.0 software (Media Cybernetics, Maryland, USA) and values with different superscript letters were significantly different from each other at *P* < 0.05. All experiments were repeated three times.

**Figure 5:**
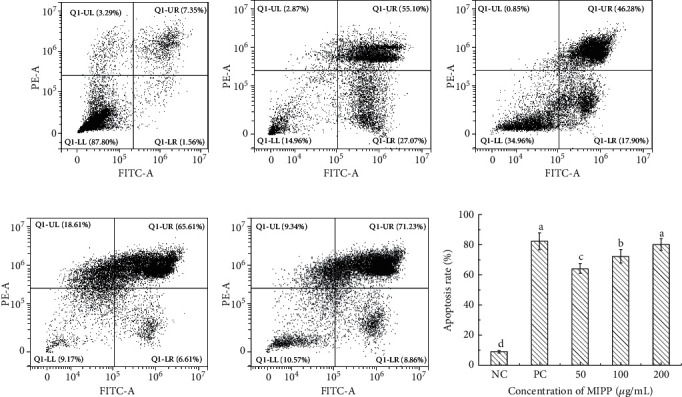
MIPP-induced HeLa cell apoptosis assessed by flow cytometry using Annexin V/PI double staining. (a) Control (untreated) cells. (b) Cells cultured with resveratrol (50 *μ*M). (c), (d), and (e) Cells treated with 50, 100, and 200 *μ*g/mL of MIPP, respectively. (f) Quantitative analysis of apoptosis rate in HeLa cells from flow cytometry. Cells were cultured for 12 h and then treated with different concentrations of MIPP and resveratrol for another 12 h. Annexin V/PI was used to stain cells and apoptosis was analyzed by flow cytometry. Values with different superscript letters were significantly different from each other at *P* < 0.05. All experiments were repeated three times.

To determine if the inhibition of cell proliferation by MIPP was due to apoptosis, morphological assays were carried out. DNA fragmentation of HeLa cells was observed by TUNEL analysis. In this analysis, 4′,6-diamidino-2-phenylindole (DAPI) excites blue fluorescence in the nuclei of cells and the presence of tetramethyl-rhodamine-5-dUTP excites red fluorescence. Thus, under the fluorescence microscope, the nuclei of apoptotic cells exhibited a light purple fluorescence. As shown in Figure 4(a), the nuclei of untreated HeLa cells exhibited blue fluorescence. After treating HeLa cells with the positive control, resveratrol, the nuclei of apoptotic cells were light purple while other cells remained blue (Figure 4(b)). After MIPP treatment, the number of cells containing light purple nuclei increased in a dose-dependent manner (Figures 4(c)–4(e)). TUNEL staining in the same samples was increased from 0.15 ± 0.01% positive area in NC group to 6.64 ± 0.59% positive area in cells treated with resveratrol (50 *μ*M). Moreover, the ratio increased to 6.65 ± 0.89% and 10.51 ± 0.76% in HeLa cells treated with 100 or 200 *μ*g/mL MIPP, respectively. To quantitatively determine the function of MIPP in inducing HeLa cell apoptosis, Annexin V/PI staining was detected by flow cytometry. As presented in Figure 5, the apoptosis rate of the control group was 8.91 ± 0.87% (Figure 5(a)). After 12 h of treatment with different concentrations of MIPP, the amount of early and late apoptotic cells rose notably in a dose-dependent manner compared to controls. The apoptosis rate of HeLa cells increased to 64.18 ± 3.25% after treatment with 50 *μ*g/mL MIPP (Figure 5(c)). Moreover, this rate was 72.22 ± 4.46% in cells co-cultured with 100 *μ*g/mL MIPP (Figure 5(d)) and 80.09 ± 3.85% in cells co-cultured with 200 *μ*g/mL MIPP (Figure 5(e)).

### 3.5. Mechanism of MIPP-Induced Apoptosis

**Figure 6:**
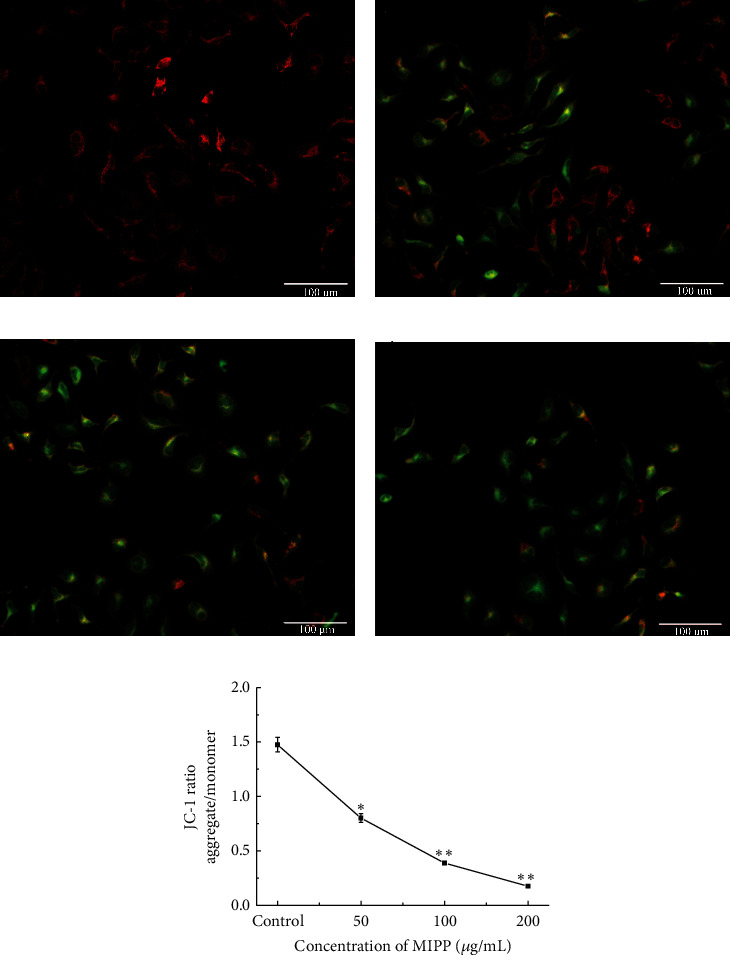
Mitochondrial membrane potential assay. The cells were seeded in a 96-well microtiter plate cultivated under standard conditions for 12 h then different concentrations (50, 100, and 200 *μ*g/mL) of MIPP were added, and cells were cultured for another 12 h JC-1 (final concentration: 2.5 *μ*g/mL per well) was added to the cells for 15 min. Intensity of fluorescence was recorded using a FACS Calibur flow cytometer. (a–d) The fluorescence immune stain of Hela cells through JC-1 method. (a) Negative control. (b–d) Different concentrations of MIPP, 50, 100, and 200 *μ*g/mL, respectively. (e) The semiquantitatively analysis for the fluorescence immune stain. Scale bar = 100 *μ*m. ^*∗*^*P* < 0.05 and ^*∗∗*^*P* < 0.01, treated versus control group by one-way ANOVA Dunnett's multiple comparison test.

**Figure 7:**
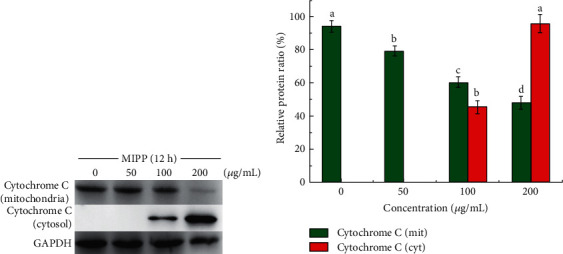
MIPP induced the release of cytochrome C from mitochondria into the cytoplasm. (a) Cytochrome C protein levels were determined by Western blot analysis. (b) The protein intensities of relative protein level were quantified from (a). The ratio represents the expression level compared with GAPDH (%). The bar represents the means ± SD; *n* = 3. Values with the same style bars with different superscript letters were significantly different from each other at *P* < 0.05. See Figures S1–S3 in the Supplementary Material for original images.

To determine whether MIPP prompted apoptosis of HeLa cells via a mitochondrial-dependent pathway, we investigated changes in mitochondrial membrane potential (Figure 6) and cytochrome C liberation (Figure 7). Normal mitochondrial membrane potential of HeLa cell was shown in red with JC-1 dimers. However, depolarized membrane potential was shown in green in JC-1 monomers (Figures 6(a)–6(d)). Results showed that following 50 *μ*g/mL MIPP treatment, the JC-1 ratio of normal cells was close to 1.5 and the ratio decreased with increasing concentrations of MIPP. When the concentration of MIPP reached 200, the JC-1 ratio was 0.80 ± 0.03, one twelfth that of the control cells. The cytochrome C content in the mitochondria and cytoplasm of HeLa cells was observed by Western blotting. After treating Hela cells with 100 ug/mL and 200 ug/mL of MIPP, the content of mitochondrial cytochrome C decreased, whereas an increase was observed in the cytoplasm. The above results indicated that MIPP induced HeLa cell mitochondrial depolarization, reduced mitochondrial membrane potential, and promoted the liberation of cytochrome C from the mitochondria to the cytoplasm.

**Figure 8:**
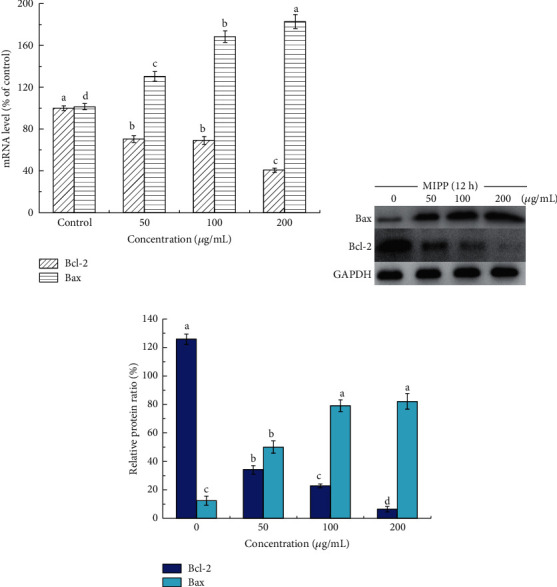
Effect of MIPP on the mRNA and protein levels of Bax and Bcl-2 in HeLa cells. (a) The mRNA levels of Bax and Bcl-2 were measured by real-time PCR. (b) Bax and Bcl-2 protein levels were determined by Western blot analysis. (c) The protein intensities of relative protein level were quantified from (b). GAPDH was selected as the internal control and the ratio represents the expression level compared with GAPDH (%). The bar represents the means ± SD. Values with the same style bars with different superscript letters are significantly different from each other at *P* < 0.05. *n* = 3. See Figures S4-S5 in the Supplementary Materials for original images.

The interactions involving proapoptotic and antiapoptotic members of the Bcl-2 protein family affected the occurrence of mitochondrial-dependent pathway apoptosis. The expression of Bcl-2 and Bax mRNA was significantly altered by 50 *μ*g/mL MIPP (Figure 8). Following treatment with 50 ug/mL MIPP, the expression Bcl-2 mRNA was reduced to 70.35% ± 3.36 of the controls while Bax mRNA increased to 130.26% ± 4.68. At this concentration of MIPP (50 *μ*g/mL), the ratio of Bcl-2/Bax was 0.54. With increasing concentrations of MIPP, the ratio decreased significantly. Specifically, the corresponding ratios of 100 *μ*g/mL and 200 *μ*g/mL MIPP were 0.40 and 0.22, respectively.

**Figure 9:**
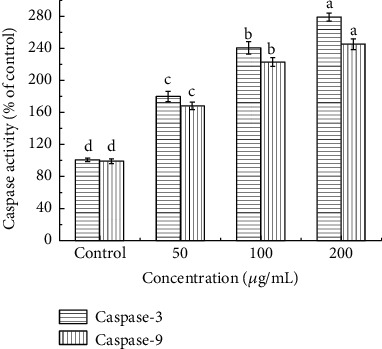
Effect of MIPP on caspase-3 and caspase-9 activities in HeLa cells. Cells were cultured for 12 h and then treated with different concentrations of MIPP for another 12 h. The activities of caspase-3 and caspase-9 were determined using a commercial kit in accordance with the instructions of the manufacturer. All experiments were repeated three times. Values with the same style bars with different superscript letters are significantly different from each other at *P* < 0.05.

**Figure 10:**
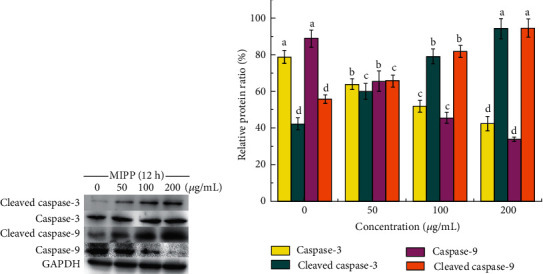
Effect of MIPP on the protein expression of caspase-3, cleaved caspase-3, caspase-9, and cleaved caspase-9 in HeLa cells. (a) The expression of caspase-3 and cleaved caspase-3 protein was identified via western blotting. (b) The protein intensities of relative protein level were quantified from (a). The ratio represents the expression level compared with GAPDH (%). The bar represents the means ± SD; *n* = 3. Values with the same style bars with different superscript letters are significantly different from each other at *P* < 0.05. See Figures S6–S11 in the Supplementary Materials for original images.

The caspase family is the main driver and primary “executor” during the apoptosis process. Thus, activities of the initiator caspase (caspase-9) as well as the effector caspase (caspase-3) were assessed (Figure 9). The results showed that in MIPP-treated cells, the activities of caspase-9 and caspase-3 increased in a concentration-dependent manner. After treating HeLa cells with 50, 100, and 200 *μ*g/mL of MIPP for 12 h, the activity of caspase-9 increased by 1.7-fold, 2.2-fold, and 2.4-fold, respectively. A similar trend was also found in the caspase-3 activity assay, which was performed using a colorimetric assay kit. When HeLa cells were cocultured with 50, 100, and 200 *μ*g/mL MIPP, caspase-3 activity increased 1.8-fold, 2.4-fold, and 2.8-fold, respectively. The expression of cleaved caspase-3 and cleaved caspase-9 was obtained through Western blotting, and results showed that MIPP promoted the expression of cleaved caspase-3 and cleaved caspase-9 in HeLa cells (Figure 10).

## 4. Discussion

Mushrooms not only have high edible and medicinal value, but are also rich in protein, so they can be used as a good source for extracting natural active peptides. The polypeptide isolated from *Pleurotus eryngii* mycelium (PEMP) was proved to have antitumor, antioxidant, and immunostimulatory activities [21]. Cordymin, a peptide extracted from the medicinal mushroom *Cordyceps sinensis*, has neuroprotective effects on rat ischemic brain [22]. The simple structure, low immunogenicity, and ease of artificial synthesis make peptides an important part of modern drug development. Usually, to separate these biologically active peptides from protein hydrolysates, a simple method is to use column chromatography, including ion exchange chromatography and/or gel filtration chromatography. The former can be separated based on the charge of ions and polar molecules, while the latter can be separated based on molecular size. However, there is a lack of standard method to extract peptide from mushroom at present. The pH of the buffer, as well as the need to add protease for enzymolysis during the extraction process, has not yet been demonstrated. Currently, peptides extracted from different kinds of mushrooms often have different extraction and purification methods. Compared with mushroom polysaccharide, which has an established and standard extraction method, the extraction of mushroom peptide needs further research.

Apoptosis is generally accompanied by obvious morphological characteristics such as nuclear condensation, dense organelles in the cytoplasm, prominent cell membranes, cell shrinkage, DNA segmentation, and the development of apoptotic fragments. The breakage of chromosomal DNA during apoptosis is a gradual and staged process. In the late stages of apoptosis, DNA is degraded into 180–200 bp fragments, and many of 3′-OH terminals are exposed that can be used to detect apoptosis by combining with certain specific dyes (such as FITC). In addition, early and late stage apoptotic cells can be identified via flow cytometry. In this study, by combining the results of TUNEL staining and flow cytometry, we determined that HeLa cells respond to MIPP treatment by entering apoptosis.

The mitochondria are the control center for the activities of cell life [23, 24]. Not only does the mitochondria serve as the center of the cell respiratory chain and oxidative phosphorylation, but they are also the center of apoptosis regulation [25]. Studies have shown that in apoptosis induced by different factors, mitochondrial membrane potential will decrease along with or even before changes in cell morphology are observed [26]. The decrease in mitochondrial membrane potential is closely related to the liberation of cytochrome C, one of the essential substances for mitochondrial initiation of the apoptotic program [27]. Many studies have reported that certain mushroom extracts can promote tumor cell apoptosis by promoting liberation of cytochrome C. For example, the *D* fraction isolated from Maitake mushrooms induced apoptosis in breast cancer cells [28]; Suillin extracted from *Suillus placidus* exhibited significant cytotoxic activities against HepG2 cells [29]; and mushroom polysaccharides isolated from *Auricularia auricular*, *Ganoderma lucidum,* and *Phellinus linteus* also induced apoptosis in human hepatoma cells [30]. The mechanisms of the above antitumor components derived from mushrooms all involved the liberation of cytochrome C.

The Bcl-2 and Bax genes are recognized as the most important regulatory genes involved in apoptosis, as they mediate the release of cytochrome C and other substances through the mitochondrial pathway. Bcl-2 and Bax proteins are located upstream of the mitochondria and are important regulatory factors in the permeability of mitochondrial membranes. Their overexpression can control the movement of cytochrome C and the triggering of the downstream caspase-3 protease, thus moderating cell survival [31]. Therefore, the ratio of Bcl-2 to Bax is considered a molecular switch for apoptosis. An increasing number of anticancer drugs can trigger the release of cytochrome C by upregulating Bax and/or downregulating Bcl-2 [32]. Our research determined that MIPP altered the balance between these Bcl-2 family members, reducing the ratio of Bcl-2/Bax, suggesting that Bcl-2 family proteins are involved in MIPP-induced HeLa cell apoptosis. These findings support the idea that MIPP-induced HeLa cell apoptosis is at least partially mediated through the mitochondrial-dependent apoptosis pathway.

It is well known that caspases play an essential role during the process of apoptosis. Apoptosis is a cascade amplification reaction process involving the irreversible limited hydrolysis of the substrate by caspases. Earlier work showed that cytochrome C liberated by the mitochondria bind to the precursor of caspase-9, triggering its activity [33]. Activated caspase-9 (initiator caspase) can trigger caspase-3 (effector caspase), which is the executor of apoptosis. Caspase-3 causes apoptosis through the hydrolysis of caspase target proteins [34]. In this study, MIPP stimulated apoptosis and regulated the ratio of Bcl-2/Bax, thereby inducing mitochondrial membrane depolarization, releasing cytochrome C, and activating caspase-3 and -9.

## 5. Conclusions

In summary, MIPP is a peptide composed of 8 amino acids obtained from the fruiting body of *Morchella importuna*. For the first time, this study established that MIPP decreased the viability of HeLa cells in a dose-dependent manner, and this decrease in cell viability was mainly caused by mitochondrial-dependent apoptosis. MIPP initiated the molecular switch controlling apoptosis via decreasing the ratio of Bcl-2/Bax, inducing mitochondrial depolarization, and decreasing membrane potential. Finally, through the release of cytochrome C, caspase-3 was activated, and apoptosis was induced. These findings revealed the molecular mechanism behind MIPP-induced apoptosis of human cervical cancer cells. In addition, its simple structure could make artificial synthesis of MIPP easy to achieve. Therefore, MIPP has potential as an antitumor drug, especially for the treatment of human cervical cancer. However, this warrants further evaluation as there are more cervical carcinoma cell lines that may be involved.

## Data Availability

The original image of the target protein was uploaded as Supplementary Materials, and all data can be obtained by contacting Dr. Pu Zhigang, the head of Biotechnology and Nuclear Technology Research Institute (http://www.chinawestagr.com/swjshjsyjs/).

## Supplementary Materials

Supplementary Materials *S1*. Original image of Cytochrome C (MIT). *S2*. Original image of Cytochrome C (cyt). *S3*. Original image of GAPDH for Cytochrome C. *S4*. Original image of Bax. *S5*. Original image of Bcl-2. *S6*. Original image of GAPDH for Bax and Bcl-2. *S7*. Original image of cleaved caspase-3. *S8*. Original image of caspase-3. *S9*. Original image of cleaved caspase-9. *S10*. Original image of caspase-9. *S11*. Original image of GAPDH for caspase.
