# Supplementary material for: Induction of Apoptosis in HeLa Cells by a Novel Peptide from Fruiting Bodies of Morchella importuna via the Mitochondrial Apoptotic Pathway
Source: Evid Based Complement Alternat Med. 2021 Aug 4;2021:5563367. doi: 10.1155/2021/5563367 (PMC8360738; doi:10.1155/2021/5563367)
Supplement: Supplementary Materials — S1. Original image of Cytochrome C (MIT). S2. Original image of Cytochrome C (cyt). S3. Original image of GAPDH for Cytochrome C. S4. Original image of Bax. S5. Original image of Bcl-2. S6. Original image of GAPDH for Bax and Bcl-2. S7. Original image of cleaved caspase-3. S8. Original image of caspase-3. S9. Original image of cleaved caspase-9. S10. Original image of caspase-9. S11. Original image of GAPDH for caspase. [file 5563367.f1.docx]

S1, Original image of Cytochrome c (MIT);


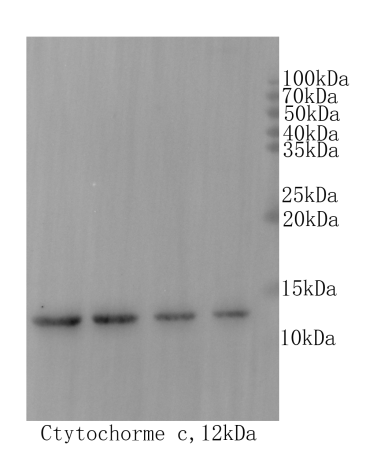


S2, Original image of Cytochrome c (cyt);


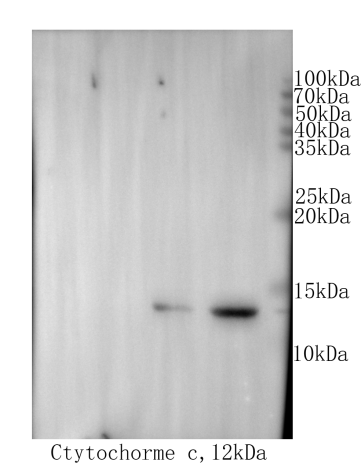


S3, Original image of GAPDH for Cytochrome c;


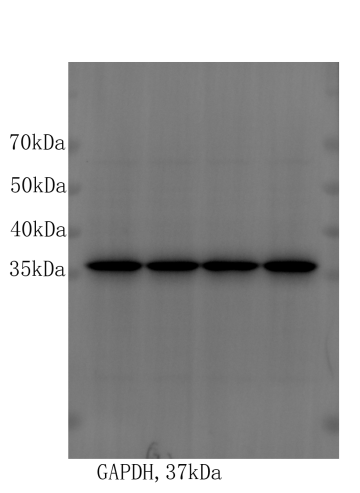


S4, Original image of Bax;


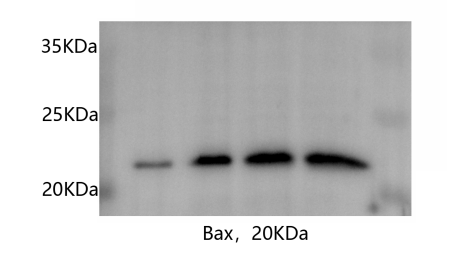


S5 Original image of Bcl-2;


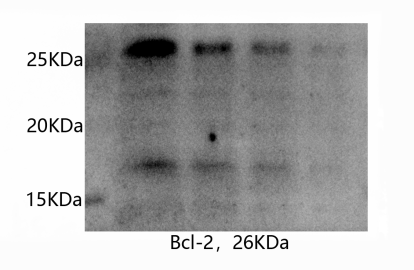


S6, Original image of GAPDH for Bax and Bcl-2;


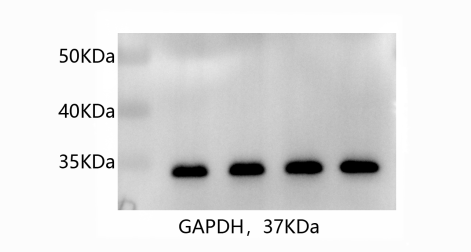


S7, Original image of cleaved-Caspase-3;


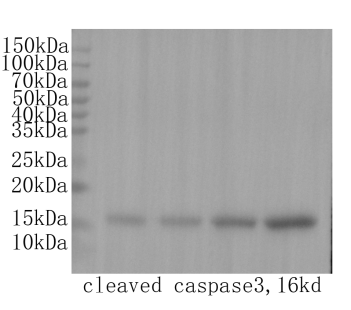


S8, Original image of Caspase-3;


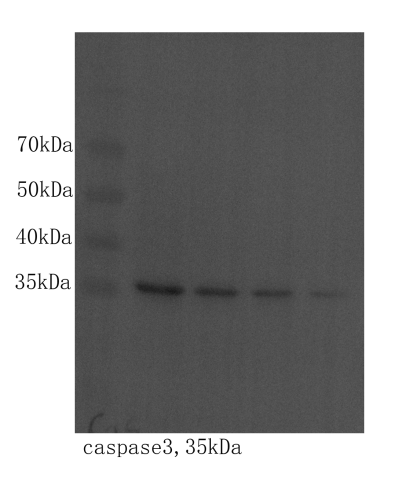


S9, Original image of cleaved-Caspase-9;


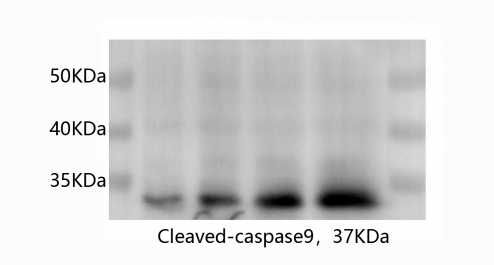


S10, Original image of Caspase-9;


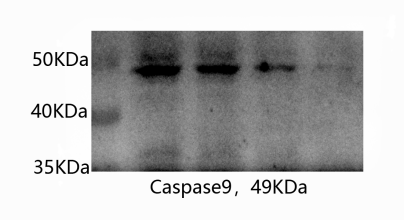


S11, Original image of GAPDH for Caspase.


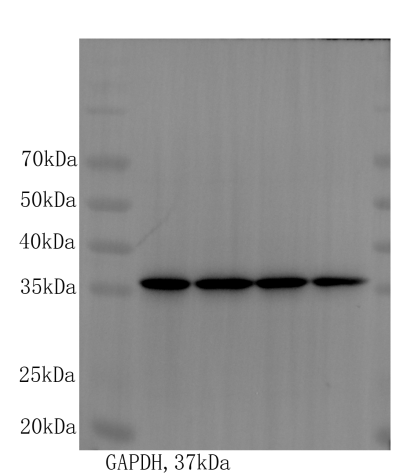


S12, The effect of MIPP on Hela, LO2 and NIH/3T3 cell proliferation. Values with different superscript letters were significantly different from each other in the same cells at *P* < 0.05.
